# Acceptance of COVID-19 vaccination and influencing factors among people living with HIV in Guangxi, China: a cross-sectional survey

**DOI:** 10.1186/s12879-022-07452-w

**Published:** 2022-05-16

**Authors:** Jinming Su, Zhenwei Jia, Xinwei Wang, Fengxiang Qin, Rongfeng Chen, Yuting Wu, Beibei Lu, Chunlin Lan, Tongxue Qin, Yinlu Liao, Minjuan Shi, Yanyan Liao, Peijiang Pan, Li Ye, Junjun Jiang, Hao Liang

**Affiliations:** 1 Biosafety Level-3 Laboratory and Joint Laboratory for Emerging Infectious Diseases in China (Guangxi)-ASEAN, Life Sciences Institute, Guangxi Medical University, Nanning, 530021 China; 2 Guangxi Key Laboratory of AIDS Prevention and Treatment, School of Public Health, Guangxi Medical University, Nanning, 530021 China

**Keywords:** HIV/AIDS, COVID-19, SARS-CoV-2, Vaccine, Acceptance

## Abstract

**Background:**

Vaccination has been proven to be an effective approach against the coronavirus disease 2019 (COVID-19) pandemic. This study aimed to determine the acceptance rate and factors influencing acceptance of COVID-19 vaccination among people living with HIV (PLWH) in Guangxi, China.

**Methods:**

A cross-sectional survey was carried out in five cities in Guangxi, China from May 7 to June 1, 2021. Questionnaires on the acceptance of COVID-19 vaccination and the related factors were conducted among PLWH recruited by simple random sampling. Univariate and multivariate logistic regression analyses were performed to identify factors associated with acceptance of COVID-19 vaccination.

**Results:**

Of all valid respondents (*n* = 903), 72.9% (*n* = 658) were willing to receive COVID-19 vaccination. Fear of severe acute respiratory syndrome coronavirus 2 (SARS-CoV-2) infection was the main reason for being willing to receive vaccination (76.0%), while the main reasons for not willing were the concerns about vaccine safety (54.7%) and the vaccination’s effect on antiretroviral therapy (ART) (50.6%). The most important factors influencing acceptance were the perception that vaccination is unsafe for HIV-infected people (aOR = 0.082, 95% CI = 0.024–0.282) and the poor efficacy in preventing SARS-CoV-2 infection in HIV-infected people (aOR = 0.093, 95% CI = 0.030–0.287). Other factors associated with acceptance included Zhuang ethnicity (aOR = 1.653, 95% CI = 1.109–2.465), highest education level of middle school, high school or above (aOR = 1.747, 95% CI = 1.170–2.608; aOR = 2.492, 95% CI = 1.326–4.682), and the vaccination having little effect on ART efficacy (aOR = 2.889, 95% CI = 1.378*–*6.059).

**Conclusions:**

Acceptance rate of the COVID-19 vaccination is relatively low among PLWH compared to the general population in China, although some patients refused vaccination due to concerns about vaccine safety and vaccination affecting ART efficacy. More research is needed to investigate the impact of the COVID-19 vaccines on ART efficacy and the effectiveness in preventing SARS-CoV-2 infection among PLWH.

**Supplementary Information:**

The online version contains supplementary material available at 10.1186/s12879-022-07452-w.

## Background

The coronavirus disease 2019 (COVID-19) pandemic has caused an enormous social and economic burden throughout the world, with more than 510.2 million COVID-19 confirmed cases and over 6.2 million deaths globally as of the end of April 2022 [1]. Currently, specific prophylactic drugs for severe acute respiratory syndrome coronavirus 2 (SARS-CoV-2) are being developed [2], and vaccination is the most cost-effective approach to slow the spread of the virus, control the outbreak, and reduce disease severity [3]. To date, more than 11.4 billion doses of vaccines have been administered worldwide, including nearly 3.3 billion doses in China [1]. Clinical trials and real-world studies have validated the effectiveness of the COVID-19 vaccines, indicating that the vaccination can effectively prevent SARS-CoV-2 infection and significantly improve multiple disease outcomes in COVID-19 patients [4, 5]. There is no doubt that vaccination is an essential measure for the full restoration of economic life [6].

Several studies have identified the high-risk factors for severe COVID-19, including elderly, hypertension, overweight, diabetes, and cardiovascular disease [7, 8], while an important population, people living with HIV (PLWH), is under-studied and should not be ignored. By the end of 2020, about 37.7 million people were reported to be living with HIV globally [9]. In China, as of October 2019, there were about 958 thousand reported HIV cases. In this country, AIDS remains the most common cause of death amongst infectious diseases, although the overall HIV epidemic remains low [10]. Some studies have shown that PLWH with combined chronic diseases had higher severity or mortality in the context of COVID-19 [11, 12]. Apparently, PLWH face the dual challenges of HIV infection and SARS-CoV-2 infection, which may lead to a greater medical burden.

Several studies have highlightened the urgency of vaccinating PLWH against COVID-19. A cohort study in South Africa reported that HIV-infected people, particularly those not receiving antiretroviral therapy (ART), were at a high risk of in-hospital death from COVID-19 and would benefit from priority vaccination [13]. A systematic review also showed that PLWH had a higher risk of infection and mortality from SARS-CoV-2 than HIV-negative individuals, suggesting that PLWH should be prioritized for COVID-19 vaccination [14]. While these studies suggest that COVID-19 vaccination should be prioritized for PLWH, large-scale data on the safety and effectiveness of COVID-19 vaccines for PLWH are still inadequate. Therefore, whether and how to choose a COVID-19 vaccine for PLWH has been a hot topic of concern. In addition, their willingness to be vaccinated is an important issue to be considered.

In this study, we selected PLWH from Guangxi, China, to investigate their COVID-19 knowledge, attitude, acceptance of COVID-19 vaccination, and the influencing factors. This study is based on the following considerations. First, the HIV/AIDS epidemic in Guangxi is still serious, with the number of HIV-reported cases ranking third in the country [15]. Second, Guangxi is located in western China and adjacent to Southeast Asia, with unique geographical environment and population diversity. Therefore, Guangxi is highly likely to suffer from the cross-border transmission and dual infection of SARS-CoV-2 and HIV [16]. Furthermore, there are currently no data on acceptance of the COVID-19 vaccination and the factors influencing acceptance for PLWH in China. This study aims to investigate acceptance and the related factors of COVID-19 vaccination among PLWH in Guangxi, and to provide vaccination strategies for this special population to respond to the COVID-19 pandemic.

## Methods

### Study design, subjects, and sampling

A cross-sectional survey was conducted in the form of face-to-face interviews from May 7 to June 1, 2021. A two-stage simple random sampling method was used in this study. Firstly, five cities (Chongzuo, Guigang, Laibin, Qinzhou, Yulin) were randomly selected from all the 14 cities in Guangxi, and then 200 participants were randomly selected from the China Information System for Diseases Control and Prevention in each city. Participants were aged 18 years or older, residing in Guangxi, confirmed HIV-1 positive, and without SARS-CoV-2 infection. A total of 1000 participants were randomly selected, and 988 questionnaires (98.8%) were collected. The final sample included 903 valid respondents (91.4%), and 85 incomplete or invalid questionnaires were excluded. All participants provided signed informed consent forms. The study was approved by the Ethics and Human Subjects Committee (EHSC) of Guangxi Medical University [No. 20210153].

### Questionnaires and data management

Based on previous research to evaluate the public’s willingness to be vaccinated [17, 18], a self-designed questionnaire was designed to obtain information on the acceptance of the COVID-19 vaccination among PLWH. A small scale preliminary investigation (60 cases) was conducted to test the validity and rationality of the questionnaire. The survey consisted of the following subsections: demographic characteristics and health status, the general status of HIV infection, knowledge of COVID-19 and current vaccines, and attitude towards COVID-19 vaccination.

All respondents were asked about their personal characteristics and health status, including age, gender, marital status, education level, occupation, ethnicity, height, weight, etc. History of severe chronic disease mainly refers to having chronic kidney disease, chronic hepatitis, diabetes, severe heart disease, chronic lung disease, etc. HIV infection information was collected from the China Information System for Diseases Control and Prevention, including the route of HIV transmission and infection, CD4^+^T cell count, infection time, ART status, adverse effects of ART, etc.

To evaluate the knowledge of COVID-19 and vaccination, participants were asked the following four questions: “What are the main ways to know about COVID-19?”, “What vaccines are available in China?”, “What age do you think is the best for COVID-19 vaccination?”, and “Which occupations should be prioritized for COVID-19 vaccination?”.

For views on COVID-19 vaccination among PLWH, six questions were asked about the factors associated with willingness to COVID-19 vaccination. The knowledge about China's vaccination policy and vaccine types was assessed with “Do you know which COVID-19 vaccines are free in China?” and “Which COVID-19 vaccine is better?”; perception of vaccine validity and safety was evaluated with “Do you think COVID-19 vaccination can help control the pandemic?” and “Do you think COVID-19 vaccines are safe?”; the influencing factors in COVID-19 vaccination among PLWH were assessed with “Is COVID-19 vaccination effective for PLWH?” and “Do you think COVID-19 vaccination affects ART efficacy?”.

The last section is to investigate attitudes toward COVID-19 vaccination. For example, the “willing to be vaccinated (WTV)”group was asked “Which COVID-19 vaccine would you like to choose?”, “Why do you want to receive the COVID-19 vaccination?”, “Would you still want to receive vaccination if the vaccine is not free?”, and “Will you still wear a mask after vaccination?”; whereas the “unwilling to be vaccinated (non-WTV)” group was asked “Why don’t you want to receive the COVID-19 vaccination?”, “Would you receive vaccination if the vaccine is free?”, and “Would you accept the COVID-19 vaccination if most people in China were already vaccinated?”.

### Statistical analysis

For analysis, we defined respondents who chose “Yes” to the question “Would you accept the COVID-19 vaccination during the pandemic in China” as the WTV group and those who chose “No” as the non-WTV group. All data were entered into EpiData software (EpiData 3.1 for Windows; The EpiData Association, Odense, Denmark), and analyzed using SPSS for Windows Version 23.0 (SPSS, Chicago, IL, USA). Descriptive statistics were performed for each variable, corresponding to specific questions in the survey, including general characteristics, knowledge of and attitude to COVID-19 and its vaccines, and reasons for accepting or refusing vaccination. The Chi-square test (*χ*^2^-test) was used for rate comparison. Univariate and multivariate logistic regression analyses were used to identify factors associated with the acceptance of COVID-19 vaccination among PLWH. Variables with statistically significant association (*P* < 0.05) with the willingness to be vaccinated were included in analyses, and the odds ratio (OR), adjusted OR (aOR), and 95% confidence interval (CI) were calculated. All statistical tests were two-sided with a significance level of *P* < 0.05.

## Results

### Demographic characteristics and HIV status

Of the 988 respondents, 903 (91.4%) completed the questionnaires and 658 (72.9%) of them were willing to receive COVID-19 vaccination to prevent SARS-CoV-2 infection. Demographic characteristics, health status, and general status of HIV infection are shown in Table 1. Among the valid respondents, 64% were male, 47.6% were 41–59 years old, 47.1% had middle school level, 46.3% had CD4^+^T cell counts of 200–500 cells/μL, 95.5% were infected through sexual transmission, 92.2% had received ART, and 11.5% had side effects of ART. Stratified analysis (Table 2) shows that there was no statistically significant difference in the effect of different CD4^+^T cell levels on willingness to be vaccinated (*P* > 0.05).

**Table 1:** Demographic characteristics of 903 subjects in Guangxi, China

Variables	Patients, No. (%) (n = 903)
Gender	
Male	578 (64.0)
Female	325 (36.0)
Age (year)	
18–40	252 (27.9)
41–59	430 (47.6)
≥ 60	221 (24.5)
Ethnicity	
Han	564 (62.5)
Zhuang	328 (36.3)
Other minorities	11 (1.2)
Occupations	
Farmer	575 (63.7)
Domestic service	139 (15.4)
Others/uncertain	189 (20.9)
Marital status	
Married	570 (63.1)
Single, divorced or widowed	326 (36.1)
Others/uncertain	7 (0.8)
Highest level of education	
Primary school or below	332 (36.8)
Middle school	425 (47.1)
High school or above	146 (16.2)
Body mass index (kg/m^2)^	
18.5–23.9	609 (67.4)
< 18.5 or ≥ 24.0	294 (32.6)
The history of serious chronic illness	
Yes	85 (9.4)
No	818 (90.6)
Transmission route	
Sexual transmission	862 (95.5)
Injecting drug abuse	33 (3.7)
Others/uncertain	8 (0.9)
CD4^+^T cell counts (cells/μL)	
< 200	131 (14.5)
200–500	418 (46.3)
> 500	334 (37.0)
Not done or not available	20 (2.2)
Infection time (year)	
< 5	394 (43.6)
5–10	357 (39.5)
> 10	152 (16.8)
ART	
Yes	833 (92.2)
No	70 (7.8)
Adverse effects of ART	
Yes	96 (11.5)
No	737 (88.5)

**Table 2:** Effects of CD4 cell count on the willingness of COVID-19 vaccine with HIV infection for 903 subjects in the survey

Variables	Patients, No.(n = 903)	*β*	S.E	OR	95% CI	*P*-Value
CD4^+^T cells count (cells/μL)						
< 250	186	Ref.				
250–349	155	0.047	0.254	1.048	0.637–1.723	0.854
350–500	208	− 0.210	0.229	0.810	0.518–1.269	0.358
> 500	334	− 0.205	0.209	0.815	0.541–1.226	0.325
Not done or not available	20	− 0.266	0.517	0.767	0.278–2.111	0.607

### Knowledge of and attitude to COVID-19 vaccination

Knowledge about COVID-19 and vaccination is shown in Table 3. Nearly half of respondents (44.7%) reported that inactivated vaccine is available in China, while 45.4% did not know the types of available vaccines in China. When surveyed on the reasons for participants’ willingness to be vaccinated (Table 4), the main reason was fear of SARS-CoV-2 infection (76.0%). The main reasons against vaccination (Table 5) were the concern about the adverse effects of vaccination (54.7%) and the concern that vaccines would affect ART efficacy (50.6%). The WTV group showed a greater preference for the inactivated vaccine (69.1%). The non-WTV group refused to accept the vaccination, even though the vaccine was free (67.3%).

**Table 3:** Knowledge of and attitude to COVID-19 vaccination among PLWH

Variables	Patients, No. (%) (n = 903)
What are the main ways to know about COVID-19?	
Phone	618 (68.4)
TV	536 (59.4)
Radio	66 (7.3)
Newspaper	39 (4.3)
Chat	314 (34.8)
Community	358 (39.6)
Others/unknown	46 (5.1)
What vaccines are available in China?	
Inactivated vaccine	404 (44.7)
mRNA vaccine	44 (4.9)
Adenovirus vaccine	19 (2.1)
Recombinant protein vaccine	26 (2.9)
Unknown	410 (45.4)
What age do you think is the best for COVID-19 vaccination? (year)	
< 18	65 (7.2)
18–40	602 (66.7)
41–59	216 (23.9)
≥ 60	102 (11.3)
Unknown	167(18.5)
Which occupations should be prioritized for COVID-19 vaccination?	
Medical personnel	671 (74.3)
Border workers	523 (57.9)
Courier/delivery/supermarket staff	360 (39.9)
COVID-19 patients/close contacts	307 (34.0)
Migrant workers	140 (15.5)
Faculty and students	222 (24.6)
Others/unknown	76 (8.4)

**Table 4:** Attitudes toward COVID-19 vaccination for WTV group

Variables	WTV Group, No. (%) (n = 658)
Which COVID-19 vaccine would you like to choose?	
Inactivated vaccine	455 (69.1)
mRNA vaccine	11 (1.7)
Adenovirus vaccine	19 (2.9)
Recombinant protein vaccine	7 (1.1)
Others	36 (5.5)
Unknown	130 (19.8)
Why do you want to receive the COVID-19 vaccination	
Fear of contracting COVID-19	500 (76.0)
Relatives/friends are willing to accept vaccine	264 (40.1)
Fear of infecting family members with COVID-19	227 (34.4)
Vaccination can improve immunity	128 (19.5)
Others	8 (1.2)
Would you still want to receive the COVID-19 vaccination if the vaccine is not free?	
Yes	457 (69.5)
No	198 (30.1)
Look at the prices	3 (0.5)
Will you still wear a mask after vaccination?	
Yes	587 (89.2)
No	29 (4.4)
Depends on the situation	42 (6.4)

WTV: willingness to vaccination

**Table 5:** Attitudes toward COVID-19 vaccination for non-WTV group

Variables	Non-WTV Group No. (%) (n = 245)
Why don't you want to receive the COVID-19 vaccination?	
Have received pneumonia/influenza (non-COVID-19) vaccine	2 (0.8)
Fear of vaccine side effects	134 (54.7)
Concern about little effect on HIV infected patients with COVID-19 vaccination	69 (28.2)
Worry that COVID-19 vaccines will affect ART efficacy	124 (50.6)
I don't think I will get COVID-19	74 (30.2)
Too expensive	11 (4.5)
Others	45 (18.4)
Would you receive vaccination if the vaccine is free	
Yes	55 (22.5)
No	165 (67.3)
Unknown	25 (10.2)
Would you accept the COVID-19 vaccination if most people in China were already vaccinated?	
Yes	68 (27.8)
No	152 (62.0)
Unknown	25 (10.2)

*Non-WTV* unwillingness to vaccination

### Factors associated with acceptance of COVID-19 vaccination among PLWH

The comparison of attitudes toward COVID-19 vaccination among PLWH is shown in Table 6 and Additional file 1. Compared with the non-WTV group, more respondents in the WTV group knew that the COVID-19 vaccine is currently free in China (*χ*^2^ = 12.531, *P* = 0.01) and thought that the vaccination is helpful or very helpful in controlling the outbreak (*χ*^2^ = 76.760, *P* < 0.001). In terms of perceptions of vaccination in relation to HIV infection, more people in the WTV group believed that COVID-19 vaccination would not affect ART efficacy compared with the non-WTV group (*χ*^2^ = 49.737, *P* < 0.001).

**Table 6:** Univariate and multivariate analysis for the influencing factors on the willingness to vaccination among PLWH

Variables	WTV Group No. (%) (n = 658)	Non-WTV Group No. (%) (n = 245)	OR (95% CI)	*P*-Value	Adjusted OR (95% CI)	*P*-Value
Ethnicity						
Han	390 (59.3)	174 (71.0)	Ref.		Ref.	
Zhuang	259 (39.4)	69 (28.2)	1.675 (1.216–2.306)	0.002	1.653 (1.109–2.465)	0.014
Other minorities	9 (1.4)	2 (0.8)	2.008 (0.429–9.389)	0.376	4.196 (0.798–22.073)	0.09
Highest level of education						
Primary school or below	207 (31.5)	125 (51.0)	Ref.		Ref.	
Middle school	328 (49.8)	97 (39.6)	2.042 (1.487–2.804)	< 0.001	1.747 (1.170–2.608)	0.006
High school or above	123 (18.7)	23 (9.4)	3.229 (1.964–5.311)	< 0.001	2.492 (1.326–4.682)	0.005
Do you think COVID-19 vaccination can help control the pandemic						
Yes, large or very large	566 (86.0)	146 (59.6)	Ref.		Ref.	
May be helpful	19 (2.9)	28 (11.4)	0.175 (0.095–0.322)	< 0.001	0.437 (0.209–0.914)	0.028
No, little or very little	5 (0.8)	4 (1.6)	0.322 (0.086–1.216)	0.095	1.851 (0.304–11.282)	0.504
Unknown	68 (10.3)	67 (27.3)	0.262 (0.178–0.384)	< 0.001	0.936 (0.532–1.647)	0.818
Do you think COVID-19 vaccines are safe?						
Yes, large or very large	562 (85.4)	129 (52.7)	Ref.		Ref.	
No, little or very little	5 (0.8)	15 (6.1)	0.077 (0.027–0.214)	< 0.001	0.082 (0.024–0.282)	< 0.001
Unknown	91 (13.8)	101 (41.2)	0.207 (0.147–0.291)	< 0.001	0.381 (0.233–0.624)	< 0.001
Do you think COVID-19 vaccination affects ART efficacy?						
Yes	37 (5.6)	29 (11.8)	Ref.		Ref.	
No	228 (34.7)	29 (11.8)	6.163 (3.312–11.466)	< 0.001	2.889 (1.378–6.059)	0.005
Unknown	393 (59.7)	187 (76.3)	1.647 (0.983–2.761)	0.058	1.432 (0.740–2.773)	0.287
Is COVID-19 vaccination effective for PLWH?						
Yes	181 (27.5)	40 (16.3)	Ref.		Ref.	
No	6 (0.9)	16 (6.5)	0.083 (0.031–0.225)	< 0.001	0.093 (0.030–0.287)	< 0.001
No difference	78 (11.9)	21 (8.6)	0.821 (0.454–1.482)	0.513	0.851 (0.427–1.697)	0.647
Unknown	393 (59.7)	168 (68.6)	0.517 (0.351–0.761)	0.001	0.785 (0.486–1.268)	0.322

In further analysis, multivariable logistic regression identified six influencing factors associated with vaccination acceptance among PLWH (Table 6): ethnicity (Zhuang *versus* Han: aOR = 1.653, 95% CI = 1.109–2.465), highest level of education (middle school versus primary school or below: aOR = 1.747, 95% CI = 1.170–2.608; high school and above *versus* primary school or below: aOR = 2.492, 95% CI = 1.326–4.682), the perception that vaccination is safe (No *versus* Yes: aOR = 0.082, 95% CI = 0.024–0.282; Unknown *versus* Yes: aOR = 0.381, 95% CI = 0.233–0.624), effectiveness of COVID-19 vaccination in PLWH (worse versus better: aOR = 0.093, 95% CI = 0.030–0.287), and whether COVID-19 vaccination affects ART efficacy (No *versus* Yes: aOR = 2.889, 95% CI = 1.378–6.059). Other factors that were not statistically significant are shown in an additional file in detail (see Additional file 1).

## Discussion

This survey is the first to investigate the acceptance of COVID-19 vaccination and influencing factors among PLWH in China. Our investigation indicated a lower acceptance rate (72.9%) of COVID-19 vaccination among PLWH compared to that in the general population (91.3%) in China [17], although the rate is higher than those in other countries such as Russia (54.85%), France (58.89%) and Sweden (65.23%) [19]. A number of studies have shown that PLWH with COVID-19 were at high risk of severe manifestations, hospitalization, and hospital mortality relative to HIV-negative persons [11, 13, 20]. Furthermore, many countries reported that the COVID-19 epidemic disrupted the delivery of HIV healthcare services, the consequences of which could increase morbidity and mortality among PLWH [21, 22]. South Africa, for example, a victim of COVID-19, has a heavy burden of HIV/AIDS. The World Health Organization estimated more than half a million additional PLWH will die in the coming year due to COVID-19 [23]. As a result, PLWH has been identified as a priority population for COVID-19 vaccination in many countries [14, 21]. Understanding the acceptance level and influencing factors of COVID-19 vaccination among PLWH is an urgent and important work, which will make the immunization of PLWH in China and the world more feasible and effective.

In this study, we found that the majority of respondents (68.4%) were aware of the COVID-19 epidemic, but a substantial proportion of respondents (45.4%) knew little about the vaccines. Compare with the non-WTV group, the WTV group had a better understanding of China’s COVID-19 vaccination policies, such as the free vaccination. Furthermore, most participants were more willing to accept inactivated vaccine, mainly because the inactivated vaccine technology is traditional and more mature [24, 25], and many studies have confirmed that inactivated vaccine has high efficacy against SARS-CoV-2 [5, 26]. Moreover, even if vaccines are not free in the future, most of the WTV group have enough incentive to pay for vaccines, which may be related to China’s vigorous implementation of COVID-19 vaccination policies and services [27]. However, some in the non-WTV group said they would refuse to be vaccinated, even if it was available for free or without epidemic limits. There is therefore an urgent need to raise awareness of the relationship between COVID-19 vaccination and HIV/AIDS to change their mindsets.

Our study also revealed that vaccine safety is a strong factor affecting vaccination acceptance, which is in line with previous studies on public concerns [28, 29]. Many real-world studies have confirmed that COVID-19 vaccines are safe and effective around the world, including inactivated vaccines and mRNA vaccines [26, 30]. The number of research above should, in theory, give the public great confidence in vaccine safety. However, this survey reflected that PLWH remains concerned about the side effects and effectiveness of COVID-19 vaccines on themselves. They should be aware that there is no live attenuated vaccines for COVID-19 in China or anywhere in the world, which may be harmful to immunocompromised people, so PLWH should be reassured about the safety of vaccine types [31, 32].

It is well known that ART is the most effective treatment available, HIV-infected patients may still be immunocompromised [33]. Regarding concerns about the impact of ART on COVID-19 vaccination, some studies have been conducted on the relationships between different ART regimens and the risk of SARS-CoV-2 infection. A multicentre study found that PLWH receiving tenofovir disoproxil fumarate (TDF)/emtricitabine (FTC) had a lower risk of COVID-19 infection compared with other ART regimens [34], and a study in South Africa had similar observations [35]. In contrast, several studies have reported that no significance in the relationship between ART regimens and COVID-19 severity in PLWH [36, 37]. However, no studies to date have focused on whether the vaccines affect ART efficacy. Our data indicated that whether vaccination affects ART efficacy was an influential factor in willingness to be vaccinated. Those who believed that the vaccination would not affect ART efficacy were more likely to be vaccinated than those who thought that the vaccination would affect ART efficacy (aOR = 2.889, 95% CI = 1.378–6.059). While whether the COVID-19 vaccine affects the effectiveness of ART is a topic that requires further research, some experts believed that prevention of SARS-CoV-2 infection is important, and recommended that PLWH on long-term antiviral therapy with a well-controlled HIV viral load and no vaccination contraindications should receive inactivated vaccine as soon as possible [38].

In addition, this study found that the higher the education level, the higher the willingness to be vaccinated, which is consistent with previous results among the general population [19, 39]. However, the majority of PLWH in Guangxi, China had low education levels [40], so it is necessary to strengthen the understanding of the COVID-19 vaccination among this population. We also observed that ethnicity was a factor influencing the acceptance of vaccination among PLWH. This is most likely because Guangxi is an ethnic minority region (mainly Zhuang Ethnicity) bordering Southeast Asia, and the frequent international trades and personnel movements are likely to increase the risk of COVID-19 infection [41], which may lead them to be more willing to be vaccinated to protect themselves.

Furthermore, a cohort study found that lower CD4^+^T cell counts were associated with COVID-19 mortality [35], however, other studies found no correlation between CD4^+^T cell counts or HIV viral load and COVID-19 outcomes [36, 42]. Our results also showed that CD4^+^T cell counts were not statistically significant for acceptance of COVID-19 vaccination by stratified and multivariate analysis. In addition, our results showed no significant difference in gender and age among PLWH related to vaccination acceptance, which is inconsistent with some studies [7, 17] and maybe attributed to the sample size of the survey.

Several limitations should be taken into account in this study. First, our survey used simple random sampling, which may lead to selection bias in some results. Second, we did not find HIV-infected people who had received the vaccine before this investigation and we knew little about the side effects of the vaccination or its effect on ART efficacy among PLWH. So we need to know more about what happens after vaccination. Follow-up studies will be conducted on SARS-CoV-2-vaccinated PLWH, including side effects of vaccination and changes in immune functions such as CD4^+^T cell counts and viral load, to provide a reference for future vaccination strategies.

## Conclusions

In summary, the study indicates a relatively low COVID-19 vaccination acceptance rate among PLWH compared to the general population in China. The safety and effectiveness of vaccines for PLWH were identified as the important factors in vaccination acceptance, which can be used to design a programme to promote vaccination among the PLWH population. Some patients refused vaccination, mainly because of concerns about vaccine safety for PLWH and vaccine’s impact on ART efficacy. Therefore, knowledge about HIV and COVID-19 vaccination, including the necessity for vaccine protection against SARS-CoV-2 should be strengthened and popularized, which could improve awareness to make better choices about vaccines and achieve the goal of herd immunity. PLWH should insist on preventive measures regardless vaccinated or not, meanwhile, more attention should be paid to treatment services for PLWH to avoid an increase in HIV-related morbidity and mortality during the COVID-19 pandemic.

## Supplementary Information

**Additional file 1: **Univariate and multivariate analysis for the other factors on the willingness to vaccination among PLWH.

## Data Availability

The HIV patient datasets generated and/or analysed during the current study are not publicly available due to ethical and legal reasons, but are available from the corresponding author on request.

## Abbreviations

COVID-19: Coronavirus disease 2019
HIV: People living with human immunodeficiency virus
AIDS: Acquired immunodeficiency syndrome
SARS-CoV-2: Severe acute respiratory syndrome coronavirus 2
ART: Anti-retroviral therapy
OR: Odds ratio
aOR: Adjusted odds ratio
CI: Confidence Interval
WTV: Willing to be vaccinated
non-WTV: Unwilling to be vaccinated
BMI: Body mass index
TDF: Tenofovir disoproxil fumarate
FTC: Emtricitabine
