# Supplementary material for: Acceptance of COVID-19 vaccination and influencing factors among people living with HIV in Guangxi, China: a cross-sectional survey
Source: BMC Infect Dis. 2022 May 16;22:471. doi: 10.1186/s12879-022-07452-w (PMC9109427; doi:10.1186/s12879-022-07452-w)
Supplement: Supplementary file 1 — Additional file 1: Univariate and multivariate analysis for the other factors on the willingness to vaccination among PLWH. [file 12879_2022_7452_MOESM1_ESM.docx]

**Additional file 1.** Univariate and multivariate analysis for the influencing factors on the willingness to vaccination among PLWH

| **Variables** | **WTV Group**  **No. (%) (n=658)** | **Non-WTV Group No. (%) (n=245)** | **OR(95%CI)** | ***P*-Value** | **Adjusted OR(95%CI)** | ***P*-Value** |
| --- | --- | --- | --- | --- | --- | --- |
| **Gender** |  |  |  |  |  |  |
| Female | 230（35.0） | 95（38.8） | Ref |  | Ref |  |
| Male | 428（65.0） | 150（61.2） | 1.179(0.871-1.595) | 0.288 | 0.835(0.572-1.218) | 0.348 |
| **Age (year)** |  |  |  |  |  |  |
| 18-40 | 204（31.0） | 48（19.6） | Ref |  | Ref |  |
| 41-59 | 308（46.8） | 122（49.8） | 0.594(0.407-0.867) | 0.007 | 0.741(0.459-1.196) | 0.22 |
| ≥60 | 146（22.2） | 75（30.6） | 0.458(0.301-0.697) | <0.001 | 0.860(0.490-1.508) | 0.598 |
| **Occupations** |  |  |  |  |  |  |
| Farmer | 407（61.9） | 168（68.6） | Ref |  | Ref |  |
| Domestic Service | 100（15.2） | 39（15.9） | 1.058(0.701-1.597) | 0.787 | 0.671(0.400-1.127) | 0.132 |
| Others/Uncertain | 151（22.9） | 38（15.5） | 1.640(1.101-2.443) | 0.015 | 0.865(0.524-1.426) | 0.57 |
| **Marital status** |  |  |  |  |  |  |
| Married | 396（60.2） | 174（71.0） | Ref |  | Ref |  |
| Single, divorced or widowed | 255（38.8） | 71（29.0） | 1.578(1.149-2.168) | 0.005 | 1.339(0.911-1.967) | 0.137 |
| Others/Uncertain | 7（1.1） | 0（0.0） | —— |  | —— |  |
| **Body Mass Index (kg/m^2^)** |  |  |  |  |  |  |
| 18.5-23.9 | 439（66.7） | 170（69.4） | Ref |  | Ref |  |
| <18.5 or >=24.0 | 219（33.3） | 75（30.6） | 1.131(0.824-1.552) | 0.447 | 1.183(0.811-1.726) | 0.382 |
| **The history of serious chronic illness** |  |  |  |  |  |  |
| Yes | 55（8.4） | 30（12.2） | Ref |  | Ref |  |
| No | 603（91.6） | 215（87.8） | 1.530(0.955-2.451) | 0.077 | 1.749(0.991-3.085) | 0.054 |
| **Transmission route** |  |  |  |  |  |  |
| Sexual transmission | 631（95.9） | 231（94.3） | Ref |  | Ref |  |
| Injecting drug abuse | 22（3.3） | 11（4.5） | 0.610(0.145-2.573) | 0.501 | 0.773(0.158-3.784) | 0.751 |
| Others/Uncertain | 5（0.8） | 3（1.2） | 0.732(0.350-1.533) | 0.409 | 0.586(0.220-1.560) | 0.285 |
| **CD4^+^T cells（cells/μL）** |  |  |  |  |  |  |
| <200 | 99（15.0） | 32（13.1） | Ref |  | Ref |  |
| 200-500 | 307（46.7） | 111（45.3） | 0.894(0.568-1.407) | 0.628 | 0.796(0.459-1.379) | 0.415 |
| >500 | 238（36.2） | 96（39.2） | 0.801(0.504-1.274) | 0.349 | 0.779(0.435-1.395) | 0.4 |
| Not done or not available | 14（2.1） | 6（2.4） | 0.754(0.268-2.125) | 0.594 | 1.277(0.370-4.403) | 0.699 |
| **Infection time (year)** |  |  |  |  |  |  |
| <5 | 279（42.4） | 115（46.9） | Ref |  | Ref |  |
| 5-10 | 261（39.7） | 96（39.2） | 1.121(0.814-1.542) | 0.484 | 1.148(0.771-1.708) | 0.496 |
| >10 | 118（17.9） | 34（22.4） | 1.431(0.922-2.219) | 0.11 | 1.326(0.749-2.345) | 0.333 |
| **ART** |  |  |  |  |  |  |
| Yes | 611（92.9） | 222（90.6） | Ref |  | Ref |  |
| No | 47（7.1） | 23（9.4） | 0.742(0.441-1.251) | 0.263 | 1.253(0.538-2.922) | 0.601 |
| **Adverse effects with ART** |  |  |  |  |  |  |
| Yes | 70（11.5） | 26（11.7） | Ref |  | Ref |  |
| No | 541（88.5） | 196（88.3） | 1.025(0.635-1.655) | 0.919 | 1.621(0.911-2.885) | 0.1 |
| **Do you know which COVID-19 vaccines are free in China?** |  |  |  |  |  |  |
| Yes | 617（93.8） | 212（86.5） | Ref |  | Ref |  |
| No | 1（0.2） | 0（0.0） | —— |  | —— |  |
| Unknown | 40（6.1） | 33（13.5） | 0.416(0.256-0.678) | <0.001 | 0.615(0.338-1.119) | 0.111 |
| **Which type of COVID-19 vaccine is better?** |  |  |  |  |  |  |
| Domestic vaccine | 492（74.8） | 110（44.9） | Ref |  | Ref |  |
| Foreign/Imported vaccine | 21（3.2） | 11（4.5） | 0.427(0.200-0.911) | 0.028 | 0.644(0.270-1.537) | 0.322 |
| No difference | 34（5.2） | 17（6.9） | 0.447(0.241-0.829) | 0.011 | 0.669(0.322-1.389) | 0.281 |
| Unknown | 111（16.9） | 107（43.7） | 0.232(0.166-0.325) | <0.001 | 0.487(0.305-0.776) | 0.002 |
